# Supplementary material for: Exploring needs and priorities in digital health management for rare disease patients and their caregivers: A mixed-methods study
Source: PLOS Digit Health. 2026 Sep 28;5(9):e0001240. doi: 10.1371/journal.pdig.0001240 (PMC13618911; doi:10.1371/journal.pdig.0001240)
Supplement: Supplementary file 1 [file pdig.0001240.s001.docx]

**Survey questionnaire (Patients)**

# Introduction

This survey is conducted by a research group at [X] in collaboration with [X].

It is intended for individuals aged 14 and older who are affected by at least one rare disease or who are caregivers of individuals affected by at least one rare disease.

We would like to learn about your needs, priorities, and concerns regarding the use of digital health technologies to improve the daily management of your rare disease or that of a person in your care.

We say digital health to mean the use of services such as:

- applications
- portals and websites
- online support groups
- online medical consultations

Or tools such as:

- mobile devices (e.g., smartphones or tablets)
- connected electronic devices such as smartwatches or medical devices connected to the Internet (e.g., connected pillbox, glucometer, connected scale)

Digital health also includes the analysis of clinical or genetic data by artificial intelligence for the purpose of improving patient care.

Digital health services and tools are a complementary solution to traditional healthcare.

The survey will take approximately 20 minutes.

# Selection criteria

1. I am 14 years old or older. YES / NO (if « YES », go to the next question. If « NO », do not continue)
   1.1. Choose one:
        1.1.1. I am 17 years old or younger
        1.1.2. I am 18 years old or older
2. Choose one:
   2.1. I am a person who has been diagnosed with at least one rare disease by a doctor or in whom at least one rare disease is suspected.
   2.2. I am a caregiver of a person affected by at least one rare disease or in whom at least one rare disease is suspected.
   2.3. None of the above (do not continue if this option is checked).

# **SECTION 1. Digital needs**

## Digital needs to inform you about your rare disease(s)

For questions 1 to 4, we want to know your interest in using digital health services and tools to **inform you about your rare disease(s)** at different stages of your medical journey. What is your interest in using digital health services and tools…

1. **To provide possible diagnoses based on your symptoms:**

| Not interested | Somewhat interested | Interested | Very interested | Already using |
| --- | --- | --- | --- | --- |

1.1. Which of the following digital services would be **most useful** for providing possible diagnoses based on your symptoms?

☐ Using an application or a web portal that utilizes your personal information.

☐ Chatting with a conversational robot (“chatbot”)

☐ Discussing on forums or social media (e.g. Facebook)

☐ Finding information on the Internet (e.g. government website, YouTube…)

☐ Meeting with a healthcare professional by videoconference (e.g. Zoom, Messenger, Teams)

☐ Emailing or chatting with a healthcare professional

2. **To inform you about the progression of your medical condition related to your disease:**

| Not interested | Somewhat interested | Interested | Very interested | Already using |
| --- | --- | --- | --- | --- |

2.1. Which of the following digital services would be **most useful** for informing you about the progression of your medical condition related to your disease?

☐ Using an application or a web portal that utilizes your personal information.

☐ Chatting with a conversational robot (“chatbot”)

☐ Discussing on forums or social media (e.g. Facebook)

☐ Finding information on the Internet (e.g. government website, YouTube…)

☐ Meeting with a healthcare professional by videoconference (e.g. Zoom, Messenger, Teams)

☐ Emailing or chatting with a healthcare professional

3. **To inform you about the risk of hereditary transmission:**

| Not interested | Somewhat interested | Interested | Very interested | Already using |
| --- | --- | --- | --- | --- |

3.1. Which of the following digital services would be **most useful** for informing you about the risk of hereditary transmission?

☐ Using an application or a web portal that utilizes your personal information.

☐ Chatting with a conversational robot (“chatbot”)

☐ Discussing on forums or social media (e.g. Facebook)

☐ Finding information on the Internet (e.g. government website, YouTube…)

☐ Meeting with a healthcare professional by videoconference (e.g. Zoom, Messenger, Teams)

☐ Emailing or chatting with a healthcare professional

4. **To better understand your disease or conditions similar to yours:**

| Not interested | Somewhat interested | Interested | Very interested | Already using |
| --- | --- | --- | --- | --- |

4.1. Which of the following digital services would be **most useful** for learning more about your disease or diseases like yours?

☐ Chatting with a conversational robot (“chatbot”)

☐ Discussing on forums or social media (e.g. Facebook)

☐ Finding information on the Internet (e.g. government website, YouTube…)

☐ Meeting with a healthcare professional by videoconference (e.g. Zoom, Messenger, Teams)

☐ Emailing or chatting with a healthcare professional

## Digital needs for managing your rare disease(s)

For questions 5 to 11, we want to know your interest in using digital health services and tools **for managing your rare disease(s)**. What is your interest in using digital health services and tools…

5. **To manage your health appointments.** For example: to make, remember, or change appointments:

| Not interested | Somewhat interested | Interested | Very interested | Already using |
| --- | --- | --- | --- | --- |

5.1. Which of the following digital services would be **most useful** for managing your health appointments?

☐ Using an application or a web portal that utilizes your personal information.

☐ Meeting with a healthcare professional by videoconference (e.g. Zoom, Messenger, Teams)

☐ Emailing or chatting with a healthcare professional

6. **To manage your medication.** For example: medication reminders, dose adjustments (pain relievers, insulin, etc.):

| Not interested | Somewhat interested | Interested | Very interested | Already using |
| --- | --- | --- | --- | --- |

6.1. Which of the following digital services would be **most useful** for managing your medication?

☐ Using an application or a web portal that utilizes your personal information.

☐ Chatting with a conversational robot (“chatbot”)

☐ Discussing on forums or social media (e.g. Facebook)

☐ Emailing or chatting with a healthcare professional

7. **To monitor symptoms or side effects that worry you after a treatment or after taking medication:**

| Not interested | Somewhat interested | Interested | Very interested | Already using |
| --- | --- | --- | --- | --- |

7.1. Which of the following digital services would be **most useful** for monitoring symptoms or side effects that worry you after a treatment or medication?

☐ Using an application or a web portal that utilizes your personal information.

☐ Chatting with a conversational robot (“chatbot”)

☐ Discussing on forums or social media (e.g. Facebook)

☐ Finding information on the Internet (e.g. government website, YouTube…)

☐ Meeting with a healthcare professional by videoconference (e.g. Zoom, Messenger, Teams)

☐ Emailing or chatting with a healthcare professional

8. **To monitor symptoms or side effects that do not worry you after a treatment or medication:**

| Not interested | Somewhat interested | Interested | Very interested | Already usin |
| --- | --- | --- | --- | --- |

8.1. Which digital services would be **most useful** for monitoring symptoms or side effects that do not worry you after a treatment or medication?

☐ Using an application or a web portal that utilizes your personal information.

☐ Chatting with a conversational robot (“chatbot”)

☐ Discussing on forums or social media (e.g. Facebook)

☐ Finding information on the Internet (e.g. government website, YouTube…)

☐ Meeting with a healthcare professional by videoconference (e.g. Zoom, Messenger, Teams)

☐ Emailing or chatting with a healthcare professional

9. **To participate in online rehabilitation programs.** For example: physiotherapy, speech therapy, etc.:

| Not interested | Somewhat interested | Interested | Very interested | Already using |
| --- | --- | --- | --- | --- |

9.1. Which of the following digital services would be **most useful** for participating in online rehabilitation programs?

☐ Using an application or a web portal that utilizes your personal information.

☐ Discussing on forums or social media (e.g. Facebook)

☐ Finding information on the Internet (e.g. government website, YouTube…)

☐ Meeting with a healthcare professional by videoconference (e.g. Zoom, Messenger, Teams)

☐ Emailing or chatting with a healthcare professional

10. **To identify clinical trials available for your disease(s) and verify your eligibility:**

| Not interested | Somewhat interested | Interested | Very interested | Already using |
| --- | --- | --- | --- | --- |

10.1. Which of the following digital services would be **most useful** for finding out about clinical trials available for your disease and check your eligibility?

☐ Using an application or a web portal that utilizes your personal information.

☐ Chatting with a conversational robot (“chatbot”)

☐ Discussing on forums or social media (e.g. Facebook)

☐ Finding information on the Internet (e.g. government website, YouTube…)

☐ Meeting with a healthcare professional by videoconference (e.g. Zoom, Messenger, Teams)

☐ Emailing or chatting with a healthcare professional

11. **To find specialist doctors or clinics for your rare disease(s):**

| Not interested | Somewhat interested | Interested | Very interested | Already using |
| --- | --- | --- | --- | --- |

11.1. Which of the following digital services would be **most useful** for finding specialist doctors or clinics for your rare disease(s)?

☐ Using an application or a web portal that utilizes your personal information.

☐ Chatting with a conversational robot (“chatbot”)

☐ Discussing on forums or social media (e.g. Facebook)

☐ Finding information on the Internet (e.g. government website, YouTube…)

☐ Meeting with a healthcare professional by videoconference (e.g. Zoom, Messenger, Teams)

☐ Emailing or chatting with a healthcare professional

## Digital needs for daily life activities

For questions 12 to 15, we want to know your interest in using digital health services and tools for **daily life activities.** What is your interest in using digital health services and tools…

12. **For support in managing your day-to-day activities.** For example: adapting your diet, your living environment, etc.:

| Not interested | Somewhat interested | Interested | Very interested | Already using |
| --- | --- | --- | --- | --- |

12.1. Which of the following digital services would be **most useful** for getting support in managing your day-to-day activities?

☐ Using an application or a web portal that utilizes your personal information.

☐ Chatting with a conversational robot (“chatbot”)

☐ Discussing on forums or social media (e.g. Facebook)

☐ Finding information on the Internet (e.g. government website, YouTube…)

☐ Meeting with a healthcare professional by videoconference (e.g. Zoom, Messenger, Teams)

☐ Emailing or chatting with a healthcare professional

13. **To maintain or improve your psychological well-being:**

| Not interested | Somewhat interested | Interested | Very interested | Already using |
| --- | --- | --- | --- | --- |

13.1. Which of the following digital services would be **most useful** for maintaining or improving your psychological well-being?

☐ Using an application or a web portal that utilizes your personal information.

☐ Chatting with a conversational robot (“chatbot”)

☐ Discussing on forums or social media (e.g. Facebook)

☐ Finding information on the Internet (e.g. government website, YouTube…)

☐ Meeting with a healthcare professional by videoconference (e.g. Zoom, Messenger, Teams)

☐ Emailing or chatting with a healthcare professional

14. **To find social services and assistance programs.** For example: resources and assistance programs in mental health, social or financial aid, specialized education services, community services, etc.:

| Not interested | Somewhat interested | Interested | Very interested | Already using |
| --- | --- | --- | --- | --- |

14.1. Which of the following digital services would be **most useful** for finding social services and assistance programs?

☐ Using an application or a web portal that utilizes your personal information.

☐ Chatting with a conversational robot (“chatbot”)

☐ Discussing on forums or social media (e.g. Facebook)

☐ Finding information on the Internet (e.g. government website, YouTube…)

☐ Meeting with a healthcare professional by videoconference (e.g. Zoom, Messenger, Teams)

☐ Emailing or chatting with a healthcare professional

15. **To talk to people who are going through the same things as you:**

| Not interested | Somewhat interested | Interested | Very interested | Already using |
| --- | --- | --- | --- | --- |

15.1. Which of the following digital services would be **most useful** for talking to people who are going through the same things as you?

☐ Using an application or a web portal

☐ Discussing on forums or social media (e.g. Facebook)

☐ Discussing with a healthcare professional by videoconference (e.g. Zoom, Messenger, Teams)

## In general:

16. **What is your interest in having digital health services and tools connected to your medical records to allow information exchange with your medical team? For example, sharing heart rate recordings.** This assumes that your medical records are electronic.

| Not interested | Somewhat interested | Interested* | Very interested |
| --- | --- | --- | --- |

17. **What is your interest in having digital health services and tools record information and, when necessary, transmit real-time alerts to your medical team?**

| Not interested | Somewhat interested | Interested* | Very interested |
| --- | --- | --- | --- |

# **Section 2. Priorities regarding the implementation of digital technologies**

For the following questions, the aim is to identify priorities related to the implementation of digital health services and tools to improve the management of rare diseases.

18. **In your opinion, what should be the priority for you?**

(A) Being informed about my rare disease(s) at different stages of my medical journey

(B) Improving the medical management of my rare disease(s)

(C) Learning to live better with my rare disease(s) on a daily basis

# **Section 3. Concerns regarding the use of digital technologies for the management of your rare disease(s)**

Regarding the use of digital health services and tools, how worried are you about the following…

19. **Your computer skills?** For example: Ability to use a technological device (smartphone, computer, tablet, etc.), ability to use the Internet, or ability to download a file.

| Not worried at all | A little worried | Somewhat worried | Very worried |
| --- | --- | --- | --- |

20. **Your information-seeking skills?** For example: ability to use a search engine such as Google.

| Not worried at all | A little worried | Somewhat worried | Very worried |
| --- | --- | --- | --- |

21. **Your access to Internet?** For example: no technological device, no Internet access at home or slow Internet speed.

| Not worried at all | A little worried | Somewhat worried | Very worried |
| --- | --- | --- | --- |

22. **Your general health knowledge?**

| Not worried at all | A little worried | Somewhat worried | Very worried |
| --- | --- | --- | --- |

23. **Protecting your personal information or privacy?**

| Not worried at all | A little worried | Somewhat worried | Very worried |
| --- | --- | --- | --- |

24. **The feeling of loss of human connection?** For example: feeling like a number rather than a person with feelings and a life outside of the illness.

| Not worried at all | A little worried | Somewhat worried | Very worried |
| --- | --- | --- | --- |

25. **Difficulty using digital health services and tools due to disability?**

| Not worried at all | A little worried | Somewhat worried | Very worried | Not applicable |
| --- | --- | --- | --- | --- |

26. **Potential costs associated with the use of digital health services and tools?**

| Not worried at all | A little worried | Somewhat worried | Very worried |
| --- | --- | --- | --- |

27. **In general, do you trust digital health services and tools to manage your rare disease(s) effectively on a daily basis?**

| No confidence | Little confidence | Some confidence | High confidence |
| --- | --- | --- | --- |

Sociodemographic and rare disease characteristics

1. **What is your gender?** By gender, we mean the current gender you identify with, which may be different from the sex assigned at birth.

☐ Female

☐ Male

☐ Other, please specify your gender: _____________________________

☐ I prefer not to answer

2. **What is your age category?**

☐ 14 to 17 years old

☐ 18 to 24 years old

☐ 25 to 34 years old

☐ 35 to 44 years old

☐ 55 to 64 years old

☐ 65 to 74 years old

☐ 75 years old and over

☐ I prefer not to answer

3. **What is the highest level of education you have completed?**

☐ No certificate, diploma, or degree (primary education or incomplete secondary education)

☐ High school diploma or equivalent (equivalency certificate)

☐ Certificate or diploma from a trade school or vocational training center (e.g., hairstyling, culinary arts, electrical work, carpentry)

☐ Certificate or diploma from a college or CEGEP

☐ Bachelor's degree or equivalent university certificate

☐ Graduate-level university certificate or diploma (post-bachelor's degree)

☐ Other, please specify: _____________________________

☐ I prefer not to answer

4. **Which of the following categories best describes your current occupation?**

Check all that apply.

☐ Full-time student

☐ Part-time student

☐ Employed, including self-employed, full-time

☐ Employed, including self-employed, part-time

☐ Unable to work

☐ Unemployed or absent from work for an extended period

☐ Retired

☐ Other, please specify: ________________________

☐ I prefer not to answer

5. **What language do you speak most often at home?**

☐ English

☐ French

☐ Other (please, specify): ________________________

☐ I prefer not to answer

6. **How long does it take you to reach the nearest healthcare center that manages your rare disease (e.g. hospital, specialized clinic)?**

☐ Less than one hour

☐ 1 to 2 hours

☐ 3 to 4 hours

☐ 5 to 6 hours

☐ More than 6 hours

☐ I prefer not to answer

7. **Have you received an official diagnosis from a doctor for one or more rare diseases?**

☐ Yes*

☐ No^¥^

**Follow-up questions for participants who answered “YES” to question 7.*

**7.1 Have you been diagnosed with more than one rare disease by a doctor?**

☐ Yes, how many: _________

☐ No

For the next questions: If you have been diagnosed with more than one rare disease, please provide information on the first rare disease for which you received an official diagnosis.

**A.7.2** **How long ago did you receive an official diagnosis for your rare disease? Please indicate the number of years.**

☐ Less than 1 year

☐ 1 to 2 years

☐ 3 to 5 years

☐ 6 to 10 years

☐ More than 10 years

☐ Since birth

(if answer to 37.2 ≠ diagnosis since birth)

**A.7.3 How many years have passed between the onset of your symptoms and the official diagnosis by a doctor?**

☐ Less than 1 year

☐ 1 to 2 years

☐ 3 to 5 years

☐ 6 to 10 years

☐ More than 10 years

(if answer to A7.2 ≠ diagnosis since birth)

**A.7.4** **How many doctors did you consult between the onset of your symptoms and the official diagnosis?**

☐ 1 to 2

☐ 3 to 5

☐ 6 to 10

☐ 11 to 20

☐ More than 20

*^¥^Follow-up questions for participants who answered “NO” to question 7.*

**B.7.1 How long ago was your rare disease first suspected by a doctor? Please indicate the number of years.**

☐ Less than 1 year

☐ 1 to 2 years

☐ 3 to 5 years

☐ 6 to 10 years

☐ More than 10 years

**B.7.2 How long ago did your symptoms first appear? Please indicate the number of years.**

☐ Less than 1 year

☐ 1 to 2 years

☐ 3 to 5 years

☐ 6 to 10 years

☐ More than 10 years

8. **Are you a person with a disability?** The term “person with a disability” is used by the Government of Quebec to refer to an individual who encounters obstacles in their daily life. It is also used to describe a disabled person.

☐ Yes

☐ No

(Only if answer to Q8 = yes)

**8.1. What type of disability do you have? Check all that apply.**

☐ Motor (partial or total impairment of mobility in the upper or lower limbs)

☐ Visual (mild, moderate, severe, or complete vision loss)

☐ Auditory (mild, moderate, severe, or complete deafness)

☐ Intellectual (significant limitations in intellectual functioning and behavior)

# Conclusion

Thank you! Your survey responses have been submitted.

If you agree to be contacted by our research team in the future to participate in a focus group related to this project, please leave your contact information by clicking on the link below.

**Important: y**our contact information will not be linked to the survey you just completed.

[LINK TO CONTACT FORM]
