# Supplementary material for: Exploring needs and priorities in digital health management for rare disease patients and their caregivers: A mixed-methods study
Source: PLOS Digit Health. 2026 Sep 28;5(9):e0001240. doi: 10.1371/journal.pdig.0001240 (PMC13618911; doi:10.1371/journal.pdig.0001240)
Supplement: Supplementary file 2 [file pdig.0001240.s002.docx]

**Discussion guide (Patients)**

# Introduction

## Team presentation

Hello and welcome everyone. We are happy to have you in our discussion group.

My name is *[X]* and I will be your facilitator today. My colleague *[X]* will take notes, manage the technology, and keep track of time.

We are part of a research group at *[X]* that focuses on health informatics.

Our team has created a research network to improve the management of rare diseases in Quebec using digital tools.

## Aim of the focus group

The purpose of today’s focus group is to understand your views on the use of digital tools and services for the management of your rare disease, and to gather your needs and priorities.

# Key details

## Meeting Flow

The focus group will last about 1 hour and 30 minutes.

First, I will explain how today’s session will work. Then, I will give a short overview of the topics we will talk about. Finally, I will ask you some questions so we can share ideas together.

## Confidentiality

We mentioned this in the consent form, but we want to remind you that today’s session will be recorded. This is to make sure we don’t miss anything and can accurately report what you say.

During the discussion, I will use your real first name, but when we write the transcript, we will replace it with a fake name. Only authorized members of the research team will have access to today’s recording. The recording will be destroyed as soon as the transcription is completed.

Conditions for success and participation rules

We invite you to share your thoughts freely on the different questions. There are no right or wrong answers. Since opinions can differ, you might not agree with others. We count on you to keep the discussion respectful.

My role is to make sure we stay focused on the topic and that everyone has a chance to speak. So, I may stop you, to invite someone else to speak or if the discussion goes off topic. It’s normal for the conversation to go in different directions. Everyone has unique experiences with their illness and the healthcare system, but I may bring the discussion back to digital tools.

Please keep your microphone and camera “on” during the discussion to make it easier to talk together. If you want to share your opinion on a topic, click the “hand” icon at the top of your screen to raise your hand when you want to speak. I will regularly go around the group so everyone can share.

If you have a technical problem during the meeting, you can write in the chat by clicking the “message bubble” icon at the top of your screen. A chat window will open so you can communicate in writing, if needed.

If you get disconnected from the meeting, simply click the purple link in the email we sent you to join again.

# Study context

We know that rare diseases bring many challenges, such as:

- Getting the right diagnosis within a reasonable time;
- Receiving proper care for your rare disease and access to the right specialists;
- Having access to effective and innovative treatments;
- Having access to reliable information about your disease.

Digital tools and services are an effective and complementary solution to traditional in-person healthcare. They can help at different stages of your care.

For example, we could have:

- Tools to make the **diagnosis** process easier. For instance, a connected medical device that tracks your blood pressure at home to help your doctor diagnose high blood pressure;
- Tools to **support shared clinical decision-making** between you and your healthcare professionals. For example, software that uses your health data to help your doctor choose the best treatment for you;
- Tools for **remote monitoring**. For example, a device that tracks your blood sugar outside the clinic and sends real-time alerts to your medical team;
- Tools to better **organize and manage your care**. For example, a digital platform that makes it easier to book medical appointments or manage your prescriptions;
- **Telemedicine** tools that allow you to receive consultations and care remotely;
- Tools to help you **manage your own care and daily activities**. For example, an app that lets you follow rehabilitation programs from home, track your medication, monitor your diet, and get suggestions adapted to your condition.

The goal of these digital tools and services is to improve your experience as a patient by giving you more control over your health and allowing you to access care from different locations.

**[Questions to participants]**

- *Do you have any questions before we start?*

# Icebreaker: General opinion on digital technologies for rare diseases

**[Questions to participants]**

- *Do you use any digital tools or services to manage your rare disease? If yes, which ones and for what reason(s)?*
- *What do you think about digital technologies for managing your health in general?*

# Digital needs assessment based on care trajectory

We will now talk about your digital needs at different stages of your rare disease journey.

## Screening

We will first discuss digital needs at the screening stage.

When we talk about screening, we mean people who do not yet have symptoms.

This includes screening tests done for newborns to detect possible rare diseases using blood and urine samples. It also includes tests for people who already have a known rare disease in their family history.

We know that screening is a crucial step for quick and effective care in the context of rare diseases.

However, we also know that you may not have gone through this step.

We would like to ask you a few questions to understand the main challenges in Quebec at the screening stage for rare diseases and how this could be improved using digital tools and services.

**[Questions to participants]**

- *In your opinion, what is the biggest challenge for screening rare diseases in Quebec?*
- *In your opinion, how could the screening process for rare diseases be improved in Quebec using digital tools?*

## Diagnosis process

Now let’s talk about the diagnostic process.

This refers to the stage that begins when a person starts showing symptoms or clinical signs.

We know that getting a diagnosis for a rare disease can be a major challenge.

The process is different for each person, but in general, it is often very long and complex.

For example:

- Many medical appointments, tests, and exams;
- Seeing multiple specialists;
- Lack of access to resources and knowledge about rare diseases;
- No proper care or even poor care due to incorrect diagnoses.

Sometimes, the search for a diagnosis leads to a diagnostic dead end, meaning it’s impossible to identify the exact cause of the disease with current knowledge and available tests.

We would like to ask you some questions to understand how the diagnostic process could be improved in Quebec using digital tools and services.

**[Questions to participants]**

- *What digital tools or services could be implemented in Quebec to make the diagnostic process easier or better?*
- *What digital tools or services would you have liked to have (or would like to have) to support you during your diagnostic process?*

## Medical management

After receiving a diagnosis, treatments and/or therapies may be needed. Follow-ups with your medical team may also be necessary to ensure proper management of your disease.

**[Roundtable with participants]**

- *What digital tools or services could help ensure better management of your rare disease?*
- *What digital tools or services would you like to use to manage episodes of instability and/or progression of your rare disease?*
- *What digital tools or services would you like to use to track symptoms or side effects that worry you after a treatment or taking a medication?*

## Daily life activities

Beyond the medical management of your rare disease, we also consider the challenges of daily life activities.

For example: moving around, eating, getting dressed, as well as managing other non-medical services like physical rehabilitation programs (physiotherapy, speech therapy…), specialized education services, psychological support services, etc.

**[Roundtable with participants]**

- *What digital tools or services would you like to use to make managing your rare disease easier on a daily basis?*

# Priorities for implementing digital technologies in Quebec

**[Roundtable with participants]**

- *Among everything we discussed today about digital needs, what would be the most important element for you; the one that should be prioritized in Quebec?*
- *Do you have any specific recommendations for implementing this priority?*

# Conclusion

This brings our meeting to an end.

Is there anything you would like to add about any of the points we discussed today?

If anyone wishes to share their perspective on any of the questions individually and anonymously, please feel free to write to the email address shown on the screen.

We sincerely thank you for the time you have given us, for your interest in this research project, and for your contribution.
