# Supplementary material for: Exploring needs and priorities in digital health management for rare disease patients and their caregivers: A mixed-methods study
Source: PLOS Digit Health. 2026 Sep 28;5(9):e0001240. doi: 10.1371/journal.pdig.0001240 (PMC13618911; doi:10.1371/journal.pdig.0001240)
Supplement: Supplementary file 3 [file pdig.0001240.s003.docx]

**Consolidated criteria for reporting qualitative studies (COREQ): 32-item checklist**

| No | Item | Description | Response |
| --- | --- | --- | --- |
| Domain 1. Research team and reflexivity | | | |
| *Personal Characteristics* | | | |
| 1 | Interviewer/facilitator | Which author/s conducted the interview or focus group? | Roxanne Dault |
| 2 | Credentials | What were the researcher’s credentials? E.g. PhD, MD | MSc |
| 3 | Occupation | What was their occupation at the time of the study? | Research coordinator |
| 4 | Gender | Was the researcher male or female? | Female |
| 5 | Experience and training | What experience or training did the researcher have? | The facilitator had formal training in qualitative research design through graduate-level coursework (MSc). She also had prior experience in planning and conducting qualitative studies, having led several projects as part of her professional career. |
| *Relationship with participants* | | | |
| 6 | Relationship established | Was a relationship established prior to study commencement? | No prior personal relationship was established between the research team and the participants before the study commenced. Recruitment was facilitated through RQMO using direct and indirect channels. All participants had previously completed an online survey administered by the research team and had voluntarily consented to be recontacted for the qualitative phase. While participants were aware that the focus groups built on the survey and addressed similar topics, no interaction with the facilitator or research team occurred beyond this initial contact prior to participation. |
| 7 | Participation knowledge of the interviewer | What did the participants know about the researcher? e.g. personal goals, reasons for doing the  research | The principal investigators (PIs) had no direct relationship with the participants (e.g., they were not involved in their care). At the start of the study, participants were provided with information about the PIs’ credentials, including their names, institutional affiliations, and funding sources. They were also informed of the specific objectives of the study, the broader aims of the research program, and the intended use of the project’s results. This information provided participants with an understanding of the researchers’ professional interest in the topic and the rationale underlying the study. |
| 8 | Interviewer characteristics | What characteristics were reported about the interviewer/facilitator? e.g. Bias, assumptions, reasons and interests in the research topic | At the beginning of each focus group, participants were informed of the facilitator’s role within the research team and the project (research coordinator). The facilitator clarified that she had no personal stake in the topics discussed and emphasized that the study objectives had been established by the principal investigators, who were not present during the sessions. She also acknowledged her role in facilitating discussions and remained attentive to potential assumptions or biases, striving to maintain a neutral and open stance throughout the data collection process. |
| Domain 2. Study design | | | |
| *Theoretical framework* | | | |
| 9 | Methodological orientation and  Theory | What methodological orientation was stated to underpin the study? e.g. grounded theory,  discourse analysis, ethnography, phenomenology, content analysis | This study was underpinned by a qualitative content analysis approach, using a hybrid (deductive and inductive) strategy. An initial coding framework was developed deductively based on findings from a preliminary online survey and applied to the focus group transcripts. This framework was iteratively refined by incorporating emergent sub-themes identified inductively from participants’ discussions. Data from all focus groups were pooled and analyzed to identify cross-cutting needs related to digital health among individuals living with rare diseases. Codes were subsequently grouped into broader themes to support an interpretive synthesis of participants’ shared priorities. This pragmatic, user-centered approach was used to generate actionable insights for digital health development. |
| *Participant selection* | | | |
| 10 | Sampling | How were participants selected? e.g. purposive, convenience, consecutive, snowball | Participants included individuals living with rare diseases and their caregivers. Recruitment was conducted in collaboration with the *Regroupement Québécois des Maladies Orphelines* (RQMO), the largest provincial organization representing individuals affected by rare diseases in Quebec. A convenience sampling strategy was used, whereby participants self-selected into the study on a voluntary basis following recruitment through the organization. |
| 11 | Method of approach | How were participants approached? e.g. face-to-face, telephone, mail, email | Participants were not approached directly by the research team. Invitations to participate were disseminated through our collaborator, the RQMO, of which participants were members. The study invitation was shared via the organization’s usual communication channels, including email newsletters, the RQMO website, and social media platforms (Facebook and LinkedIn), allowing interested individuals to volunteer for participation. |
| 12 | Sample size | How many participants were in the study? | All individuals who expressed interest in participating in a focus group were included. A total of four focus groups were conducted, each comprising 3 to 5 participants. Small group sizes were intentionally chosen to facilitate in-depth discussion and allow each participant to fully express their perspectives. In total, 15 individuals participated in the qualitative component of the study. |
| 13 | Non-participation setting | How many people refused to participate or dropped out? Reasons? | Of the 27 individuals who initially provided their contact information to be recontacted for the focus groups, 12 did not ultimately participate. Among these, 6 were unable to provide availability that aligned with other participants or the research team. The remaining 6 withdrew shortly before the sessions due to various personal reasons, including family obligations, last-minute medical appointments, or lack of response. |
| *Setting* | | | |
| 14 | Setting of data collection | Where was the data collected? e.g. home, clinic, workplace | Focus groups were conducted online via the Microsoft Teams platform. This approach was chosen because participants were geographically dispersed across different regions of the province of Quebec, enabling the inclusion of individuals from remote areas. Additionally, people living with rare diseases often face physical limitations that may restrict their ability to travel. These considerations informed the decision to conduct data collection virtually. |
| 15 | Presence of non-participants | Was anyone else present besides the participants and researchers? | Only the moderator (the research coordinator leading the project) and a research professional (responsible for note-taking and technical management of the Teams platform) were present during the focus groups. The principal investigators were intentionally not present to minimize potential acquiescence and social desirability biases, and to avoid influencing participants’ responses. |
| 16 | Description of sample | What are the important characteristics of the sample? e.g. demographic data, date | All participants included in the qualitative component were members of the *Regroupement Québécois des Maladies Orphelines* (RQMO) and were either individuals diagnosed with a rare disease or experiencing diagnostic odyssey. For the focus group involving caregivers, participants were required to be supporting a person living with a rare disease, with no restrictions on the nature of the relationship (e.g., family member or friend). No restrictions were applied regarding the type of rare disease, as the study aimed to identify shared needs and priorities across the rare disease community rather than focusing on a specific diagnosis. Participants were geographically diverse, representing various cities and regions across the province of Quebec, including both major urban centers (e.g., Montréal) and more remote areas with limited access to services. |
| *Data collection* | | | |
| 17 | Interview guide | Were questions, prompts, guides provided by the authors? Was it pilot tested? | The interview guide was developed by the research team to address the study objectives. Although the guide was not pilot tested, it was reviewed by two patient partners from the RQMO (rare disease community) to ensure the relevance and clarity of the questions. Most of the interview questions were informed by the findings of the previously conducted survey (phase 1 of this study), with the aim of further exploring and elaborating on the quantitative results. |
| 18 | Repeat interviews | Were repeat interviews carried out? If yes, how many? | In total, four “identical” focus groups were held: three with individuals living with rare diseases and one with caregivers of individuals with rare diseases. The same discussion guide was used across all groups; however, the wording was slightly adapted for the caregiver group to ensure relevance to their perspective, without changing the nature of the questions (e.g., “to support you in managing your rare disease” vs. “to support you in managing your loved one’s rare disease”). |
| 19 | Audio/visual recording | Did the research use audio or visual recording to collect the data? | All online focus groups were audio- and video-recorded. Audio recordings were transcribed verbatim for analysis. Video recordings were primarily used to capture contextual and non-verbal cues (e.g., participants nodding) to enrich and complement the verbatim transcripts. |
| 20 | Field notes | Were field notes made during and/or after the interview or focus group? | No formal field notes were taken during or after the focus groups. All sessions were audio- and video-recorded and transcribed verbatim, and the recordings were considered sufficient to capture both verbal content and relevant non-verbal cues.  In addition, each focus group was followed by a debriefing discussion with the broader research team to reflect on key elements raised during the discussions. These exchanges helped inform the analytical process. However, no modifications were made to the interview guide between focus groups in order to ensure that all participants were asked the same questions. |
| 21 | Duration | What was the duration of the interviews or focus group? | All focus groups lasted approximately 90 minutes. In each session, the entire discussion guide was covered, and all participants were given the opportunity to share their perspectives on each question. Smaller group sizes were intentionally chosen to facilitate participation and ensure that all voices could be heard. |
| 22 | Data saturation | Was data saturation discussed? | Although data saturation was not formally monitored during data collection, all four focus groups were completed prior to analysis. During thematic analysis, themes were found to be recurrent across groups, with no substantially new themes emerging in the later discussions, suggesting that data saturation was reached. |
| 23 | Transcripts returned | Were transcripts returned to participants for comment and/or correction? | Transcripts were not returned to participants for comment or correction. All transcripts were produced verbatim from the audio recordings (word-for-word), ensuring that participants’ contributions were accurately captured without alteration. |
| Domain 3. Analysis and findings | | | |
| *Data analysis* | | | |
| 24 | Number of data coders | How many data coders coded the data? | One member of the research group (the focus group moderator) conducted the initial coding of all transcripts. The coding framework (thematic nodes) was subsequently reviewed by one of the principal investigators (Anita Burgun). In addition, biweekly meetings with all research team members were held to discuss emerging issues during the coding process and to refine themes and their classification as needed. |
| 25 | Description of the coding tree | Did authors provide a description of the coding tree? | A coding tree was developed a priori using a deductive approach based on the interview guide questions. A coding manual was also created, including definitions for each node to support consistent coding of the transcripts. The coding framework remained iterative, however, and was refined throughout the analysis to incorporate new themes emerging from the data. |
| 26 | Derivation of themes | Were themes identified in advance or derived from the data? | Both. As described above, a hybrid approach was used: an initial deductive coding framework was developed based on the interview guide, and additional nodes were added inductively as new themes emerged from the data. This combined deductive-inductive approach is consistent with established thematic analysis methods, which allow for both theory-driven and data-driven theme development (Braun & Clarke, 2006). |
| 27 | Software | What software, if applicable, was used to manage the data? | NVivo software (version 14) was used to manage and analyze the qualitative data, including coding of the focus group transcripts and thematic analysis. |
| 28 | Participant checking | Did participants provide feedback on the findings? | Participants did not provide feedback on the study findings, as transcripts or results were not returned to participants for comment. |
| *Reporting* | | | |
| 29 | Quotations presented | Were participant quotations presented to illustrate the themes / findings? Was each quotation identified? e.g. participant number | Multiple participant quotations were presented for each theme in the article. Efforts were made to reflect the perspectives of all participants. Each quoted extract was identified using a participant number. |
| 30 | Data and findings consistent | Was there consistency between the data presented and the findings? | Yes, there was consistency between the data presented and the findings. Themes were grounded in the data and supported by representative participant quotations, ensuring that the interpretations accurately reflected participants’ perspectives. |
| 31 | Clarity of major themes | Were major themes clearly presented in the findings? | Yes, major themes were clearly presented in the findings. The results were organized into well-defined themes that aligned with the study objectives, which aimed to identify the needs and priorities of individuals living with rare diseases and their caregivers regarding digital tools and services to support disease management across different stages. |
| 32 | Clarity of minor themes | Is there a description of diverse cases or discussion of minor themes? | Yes, minor themes were described. In addition to the major themes, minor themes capturing less prominent but relevant needs and priorities that emerged from the discussions were also presented. These were clearly organized into defined subsections within the findings. |

## References

Braun, V., & Clarke, V. (2006). *Using thematic analysis in psychology*. Qualitative Research in Psychology, 3(2), 77–101.
