# Supplementary material for: Exploring needs and priorities in digital health management for rare disease patients and their caregivers: A mixed-methods study
Source: PLOS Digit Health. 2026 Sep 28;5(9):e0001240. doi: 10.1371/journal.pdig.0001240 (PMC13618911; doi:10.1371/journal.pdig.0001240)
Supplement: Supplementary file 4 [file pdig.0001240.s005.docx]

## RESPONSES TO THE JOURNAL’S AND REVIEWERS’ COMMENTS

## Journal requirements:

| **Journal requirements** | **Authors’ responses** |
| --- | --- |
| 1. Please amend your detailed Financial Disclosure statement. This is published with the article. It must therefore be completed in full sentences and contain the exact wording you wish to be published. i. Please clarify all sources of financial support for your study. List the grants, grant numbers, and organizations that funded your study, including funding received from your institution. Please note that suppliers of material support, including research materials, should be recognized in the Acknowledgements section rather than in the Financial Disclosure. | Modifications have been made to the “Financial Disclosure” section to provide more detailed information on the funding sources (including holders, start dates, the exact name of the funding, and the funding organizations). However, neither of the project’s funding sources has an associated grant number.  Please refer to lines 901-915 (pages 42-43) in the track changes version of the manuscript to review the corrections made. |
| ii. State the initials, alongside each funding source, of each author to receive each grant. For example: "This work was supported by the National Institutes of Health (####### to AM; ###### to CJ) and the National Science Foundation (###### to AM)." | The initials of the authors associated with each of the cited funding sources have been added, as requested.  Please refer to lines 901-915 (pages 42-43) in the track changes version of the manuscript to review the corrections made. |
| iii. State what role the funders took in the study. If the funders had no role in your study, please state: “The funders had no role in study design, data collection and analysis, decision to publish, or preparation of the manuscript.” | As requested, a statement has been added to the “Financial Disclosure” section of the manuscript to clarify the role of the funders in the project.  Please refer to lines 913-915 (page 43) in the track changes version of the manuscript to review the corrections made. |
| iv. If any authors received a salary from any of your funders, please state which authors and which funders. | None of the authors received salary support from the funding sources associated with this study. This information has now been explicitly stated in the manuscript.  Please refer to lines 914-915 (page 43) in the track changes version of the manuscript. |
| 2. We note that your Data Availability Statement is currently as follows: The survey questionnaire and the discussion guide used for the focus groups are available as Supporting information files. All data generated or analyzed during this study are included in this published article.   Please confirm at this time whether or not your submission contains all raw data required to replicate the results of your study. Authors must share the “minimal data set” for their submission. PLOS defines the minimal data set to consist of the data required to replicate all study findings reported in the article, as well as related metadata and methods (<https://journals.plos.org/plosone/s/data-availability#loc-minimal-data-set-definition>).    For example, authors should submit the following data:   - The values behind the means, standard deviations and other measures reported; - The values used to build graphs; - The points extracted from images for analysis.   Authors do not need to submit their entire data set if only a portion of the data was used in the reported study.  If your submission does not contain these data, please either upload them as Supporting Information files or deposit them to a stable, public repository and provide us with the relevant URLs, DOIs, or accession numbers. For a list of recommended repositories, please see <https://journals.plos.org/plosone/s/recommended-repositories>.  If there are ethical or legal restrictions on sharing a de-identified data set, please explain them in detail (e.g., data contain potentially sensitive information, data are owned by a third-party organization, etc.) and who has imposed them (e.g., an ethics committee). Please also provide contact information for a data access committee, ethics committee, or other institutional body to which data requests may be sent. If data are owned by a third party, please indicate how others may request data access. | Due to ethical restrictions related to participant privacy, the raw data cannot be made publicly available. Data will be available upon reasonable request from the corresponding author and with approval from the research ethics committee.  This clarification has been included in the “Data Availability” section of the article.  Please refer to lines 891-896 (page 42) in the track changes version of the manuscript to review the corrections made. |
| 3. Please ensure that your Ethics Statement is available in its entirety at the beginning of your Methods section, under a subheading 'Ethics Statement'.   If the reviewer comments include a recommendation to cite specific previously published works, please review and evaluate these publications to determine whether they are relevant and should be cited. There is no requirement to cite these works unless the editor has indicated otherwise. | As requested, the Ethics Statement section has been relocated to the beginning of the Methods section, and it has been renamed appropriately.  Please refer to lines 128-135 (pages 6-7) in the track changes version of the manuscript to review the corrections made. |
|  | Note that affiliation #4 for author Anita Burgun has been modified.    Affiliation #5 has also been added for author Christina Khnaisser.  See the first page of the manuscript. |

## Reviewers’ comments

### Reviewer #1:

| **Reviewer 1 comments** | **Authors’ responses** |
| --- | --- |
| **Summary** | |
| The authors present a mixed-methods study (149 survey respondents, 15 focus group participants) analyzing what rare disease patients and caregivers want from digital health tools in Quebec. The community wants centralized data, symptom tracking, and better communication with clinicians. The paper shows why generic health apps do not work for this population.  In my comments below, I make some suggestions of what to include. The authors are free to include them or not, the intention is to be helpful not to constrain them to a particular view. I hope that they are useful because I think this should be accepted/published, subject to minor changes. | N/A |
| **Major comments** | |
| 1. **Sample Bias and Response Rates:** The 90% female sample is expected, but warrants brief acknowledgement and discussion, because it's socially important. Sociologically, women carry a disproportionate caregiving burden for complex needs, and female patients generally experience longer diagnostic odysseys, often facing "medical gaslighting". This drives them to advocacy groups like RQMO. The authors should state how these systemic drivers create bias, and note how it affects findings on digital literacy or trust. Do the authors have a response rate? Knowing whether 149 respondents came from 300 or 3,000 invitations would clarify the engagement level. | We agree with the reviewer. Only volunteers participated in the study and the participation rates may reflect population engagement in the domain. We have followed the reviewer’s recommendation and added a sentence in the “Study limitations and strengths” section.  Please refer to lines 839-859 (page 40) in the track changes version of the manuscript to see this addition. |
| 2. **Alert Fatigue and Behavioral Economics:** The mention of "alert fatigue" (Lines 688-691) identifies a common failure point. From a behavioral economics perspective, clinicians facing a flood of raw, patient-generated symptom data will discount or ignore it due to cognitive overload. This friction prevents digital health adoption. Patient-centered tools only work if they are aggressively filtered to be clinician-friendly. The discussion needs to reflect this. | We would like to thank the reviewer for pointing out the potential negative impact associated with the flood of raw data sent by patients. Rare disease patients would like health professionals to get real-time access to their data and make decisions based on this data. We agree that this could be a source of failure.  We have added a paragraph in the Discussion Section just after the sentence “However, such an approach raises concerns about potential “alert fatigue” among clinicians managing large patient populations.”  Please refer to lines 724-731 (page 35) in the track changes version of the manuscript to see this addition.  However, to our knowledge, no study has been published yet about the rare disease domain and patient-generated data sent to clinicians. Moreover, as suggested by other authors working on monitor devices, alert burden may be reduced by incorporating prediction AI models for alarm triage (e.g. Vetter et al., 2024; Drew et al., 2014; Hu et al., 2019). Similar options should be explored before implementing digital solutions in our domain.  Vetter J, Lim K, Dijkstra TMH, Dargaville PA, Kohlbacher O, Macke JH, et al. Neonatal apnea and hypopnea prediction in infants with Robin sequence with neural additive models for time series. PLOS Digit Health. déc 2024;3(12):e0000678. doi:10.1371/journal.pdig.0000678 PubMed PMID: 39671454; PubMed Central PMCID: PMC11642933.  Drew BJ, Harris P, Zègre-Hemsey JK, Mammone T, Schindler D, Salas-Boni R, et al. Insights into the problem of alarm fatigue with physiologic monitor devices: a comprehensive observational study of consecutive intensive care unit patients. PLoS One. 2014;9(10):e110274. doi:10.1371/journal.pone.0110274 PubMed PMID: 25338067; PubMed Central PMCID: PMC4206416.  Hu X. An algorithm strategy for precise patient monitoring in a connected healthcare enterprise. NPJ Digit Med. 30 avr 2019;2:30. doi:10.1038/s41746-019-0107-z PubMed PMID: 31304377; PubMed Central PMCID: PMC6550269. |
| 3. **Privacy and Self-Selection:** The conclusion that rare disease patients are willing to share data (only 50% expressed privacy concerns) is likely overstated. Recruiting through advocacy networks yields a highly activated cohort desperate for solutions. This group self-selects for higher trust in data sharing compared to the general population. I'm not arguing with the authors, and this is my view. If the authors think differently, it might be worth suggesting something like, "a reviewer suggested X, however...". What that does is demonstrate that there isn't consensus in the interpretation and in my view, adds value as a potential avenue for further exploration. For what it's worth, I reckon a discrete choice experiment could be really useful and interesting to undertake here, and although I'm not a native French-speaker, if the authors are interested in further advice/suggestions on this, I'm happy to provide them. | We agree with the reviewer that we cannot exclude potential inherent bias, and we re-wrote this section in the manuscript.  Please refer to lines 775-793 (pages 37-38) in the track changes version of the manuscript to see these modifications. |
| 4. **Generalizability (The Quebec Context):** Quebec's RAMQ system provides universal public insurance but struggles with geographic bottlenecks and wait times (common to many social health systems). Patients are fighting systemic access barriers rather than financial ones. The findings generalize best to similar universal, geographically dispersed systems--such as the UK NHS, Australia's Medicare, or New Zealand-- rather than multi-payer environments like the US. This is important, because under-recognized is both access difficulties and catastrophic health expenditure for people with rare diseases. | We would like to thank the reviewer for his/her comment on generalizability. We have added a paragraph in the “Study Limitations and Strengths” section to reinforce the notion that responses are dependent on the healthcare system but that results may be a starting point for establishing priorities.  Please refer to lines 860-869 (pages 40-41) in the track changes version of the manuscript to see this addition. |
| **Minor comments** | |
| **Line 284:** The qualitative quote mentioning "20-year wait times" is compelling. Mention this in the introduction to frame why the diagnostic odyssey requires digital intervention. I'm tempted to provide a quote to one of my own papers here, but on principle I won't. There's plenty of great papers describing wait times. The typically quoted figure is somewhere around years. I don't know how to interpret the "20-year" experience, but I feel like it might be important in the future for us to come back to this. | Thank you for the reviewer’s comment.  In the Introduction Section: The phenomenon of diagnostic odyssey, as well as several factors that may contribute to prolonged time to diagnosis, are already discussed in the Introduction section (lines 63-69, page 4). As this background information provides the context needed to interpret the findings presented in the Discussion, we did not consider additional details necessary and therefore made no further modifications to the manuscript.  In the Discussion Section: We added three references in the corresponding paragraph in the Discussion section, first to highlight the patients’ perception (Nguyen et al., 2025), and then to cite some authors who also believe that AI and digital tools will play a central role to address that issue. One publication is more from the technology domain (Ho et al., 2026) and the other reference was published in 2026 in Human Genomics (Li et al., 2026).  Please refer to lines 815-816 (page 39) in the track changes version of the manuscript to see these additions. |
| **Line 364 (AI):** Participants suggesting AI for symptom pattern recognition is topical. Briefly discuss how patients currently use LLMs for self-diagnosis, and the clinical risks involved. You might (I'm not sure), identify a gap in the literature around this. This group might be well placed to research that, but in either case, it signposts for future researchers, making this paper an important citation in work like this. | We thank the reviewer for his/her comment on the role that LLM could play in the future. We agree with that. We have mentioned LLM and generative AI in the section on “modern digital tools and state-of-the-art technologies”.  As literature about such tools has grown dramatically, we have added a recent review article published in 2026 as new reference (Feng et al., 2026) and we have added a sentence about Feng’s conclusion that four main areas of research be developed concerning AI-based chatbots: *text quality, clinical efficacy, user engagement, and safety*.  Please refer to lines 761-763 (page 37) in the track changes version of the manuscript to see this addition. |
| **Conclusion** | |
| This is a strong paper providing useful data in the digital health space. Hopefully it's evidence that I enjoyed it and I appreciate that the work was done. The mixed-methods approach captures the specific frustrations of the rare disease community.  Addressing the drivers of demographic bias, adjusting the privacy findings, and expanding on the behavioral friction of clinician alert fatigue will improve the manuscript. I recommend minor revision.  Note to the editorial team: If accepted, I advise promoting this piece. It will draw attention from pharma/lifesciences groups, the rare disease community, and organizations like the World Economic Forum, which have an active interest in rare disease support and digital management. | N/A |
| **Reporting Checklist Assessment (COREQ)**  Recommendation: The authors should review COREQ or SRQR standards and clarify the missing qualitative methodology details (particularly saturation, non-participation, and participant checking). If you download either/both from Equator, assess against this, implement the improvements, then include the checklist as an attachment. They can be incorporated into a supplement and don't need to be in the manuscript. What this does is increase the assessed quality, particularly for things like meta-analyses, and this increases the likelihood of citation/impact. I suspect (my view), that increasingly as AI-assisted manuscript review comes in, that checklists will be considered standard. I've asked for them for many years, but this context makes it even more important and worth the additional work.  A cursory independent assessment against the 32-item COREQ checklist shows:   - Reported: Methodological orientation, participant selection via advocacy groups, setting (online), sample demographics, data coder count, and quotation presentation. - Missing/Incomplete:   - Research Team: Specific credentials, occupation, and gender of the facilitator; participant knowledge of the interviewer.   - Study Design: Data saturation, rates of non-participation/drop-outs for the focus groups, and whether transcripts were returned to participants.   - Analysis: Formal participant checking of the findings or quotes. | We thank the reviewer for this insightful suggestion. The 32-item COREQ checklist has been completed and incorporated as supplementary material. To prevent further extending an already lengthy manuscript, additions to the main text have been kept to a minimum. Nonetheless, the COREQ items have been addressed in detail to ensure greater transparency in reporting the qualitative methodological approach of our study (e.g., regarding data saturation, non-participation, participant checking, etc.).  Please refer to the newly added Supplementary material S3 (32-item COREQ checklist) and to lines 223-224 (page 10) in the track changes version of the manuscript for a detailed review of the revisions. |

### Reviewer #2:

| **Reviewer 2 comments** | **Authors’ responses** |
| --- | --- |
| **Summary** | |
| This study examines the role of digital health technologies in addressing the complex challenges faced by individuals living with rare diseases and their caregivers, including barriers to diagnosis, treatment access, and care coordination. The study addresses a highly relevant and underexplored area, and its mixed-methods approach has the potential to provide valuable, practice-oriented insights into the needs and priorities of rare disease patients and their caregivers.  That said, there are several areas where further clarification and elaboration would strengthen the manuscript and enhance its overall contribution: | N/A |
| **Comments** | |
| In the methods section, the authors state that RQMO members were recruited as participants. However, readers who are not familiar with this organization may lack sufficient background to understand the sampling choice. It would be helpful to briefly describe what RQMO is, the characteristics of its members, and the rationale for targeting this group. In addition, the authors should discuss the strengths and limitations associated with this sampling strategy. | At the reviewer’s request, we have provided a more detailed description of the RQMO in the manuscript to offer readers a clearer understanding of this population.  Please refer to lines 145-156 (pages 7-8) in the track changes version of the manuscript for a detailed review of the revisions. |
| Based on the data analysis and results sections, the quantitative survey appears to be used primarily for descriptive purposes, which somewhat limits the implications of the findings. Given the context of rare disease patients and their caregivers, one might expect more exploratory insights into their motivations and needs regarding telemedicine or digital health tools. However, participants were restricted to selecting from predefined options, which may have constrained the depth of responses. This raises a broader question about how the sequential mixed-methods design was intended to function.  Specifically, what aspects were meant to be addressed by the survey, and what additional insights were expected from the interviews? The complementary roles of the two methods should be clarified. | The objective of our study was described as follows in the Introduction section: “Specifically, we aimed to: (i) describe interest and current use of digital health tools among individuals with rare diseases and their families; (ii) identify and prioritize patient-defined needs and preferences for patient-centered digital health solutions; and (iii) examine perceived challenges and conditions for successful implementation. By combining survey data with focus group insights, this study extends prior findings by offering a broader and more actionable understanding to inform the development and deployment of tailored digital health tools for the rare disease community.”  As such, our aim was to explore the roles that people living with rare diseases expect from digital tools, and what are their priorities. Participation in the survey was established on a voluntary basis. We conducted interviews to get more in-depth representation of what this group of patients and caregivers consider priorities and why they do so. We found their input useful to provide recommendations or, at least, support for discussion, to the IT/AI communities, to the health professionals, and to the decision makers. |
| It is also unclear why caregivers were asked to respond from the perspective of the individuals they care for, rather than from their own viewpoints. Caregivers may have distinct needs and experiences, and capturing these directly could provide valuable insights. The authors should justify this design choice. | We agree with Reviewer 2 that caregivers may have distinct needs. However, our objective was to identify priorities for rare disease patients, and we asked them to answer from the viewpoint of the individual they were caring for. As we focused on rare diseases with genetic causes, caregivers are usually parents of children living with rare diseases.  In the Results Section, sub-section “Digital health needs for rare diseases”, we have replaced the sentence: “Results from rare disease patients and caregivers were combined, and no subgroup analysis was conducted as caregivers were asked to answer from the viewpoint of the individual they are caring for.” by: “As in common in the rare disease context, where caregivers are typically parents caring for a child with a genetic disorder, caregivers were asked to respond from the perspective of the individual for whom they provided care. Responses from patients with rare diseases and caregivers were combined, and no subgroup analyses were conducted.”  Please refer to lines 272-277 (page 15) in the track changes version of the manuscript for a detailed review of the revisions. |
| Finally, the importance of telemedicine for rare disease patients and their caregivers could be better articulated. How does telemedicine use in this population differ from what has been reported in the broader literature on general patient populations? A more explicit comparison in both the introduction and discussion sections would help to strengthen the study’s contribution. I wish the authors the best in further refining and developing this work for publication. | We thank the reviewer for this comment. As suggested, we have added a sentence in the Introduction Section: “Concretely, given the high number of rare genetic disorders and their very low prevalence, only a few medical experts possess extensive knowledge of specific diseases, and patients have to be followed up in rare disease excellence centers, leading to significant challenges in accessibility, logistics, and continuity of care.”  Please refer to lines 83-86 (page 5) in the tracked changes version of the manuscript for a detailed review of the revisions.  Moreover, in the Discussion Section, we have compared our result to what is advocated by rare disease organizations in the United States and in Europe.  Please refer to lines 709-718 (page 35) in the track changes version of the manuscript for a detailed review of the revisions. |

## Reviewer #3:

| **Reviewer 3 comments** | **Authors’ responses** |
| --- | --- |
| **Summary** | |
| Understanding the challenge of consolidating data from rare diseases, I highly commend the efforts undertaken by the team. While the dataset is small (n = 15 for the follow-up activities), the 150+ respondents can compensate for this. | N/A |

### Other requirements

| **Figure resubmission:** While revising your submission, we strongly recommend that you use PLOS’s NAAS tool (https://ngplosjournals.pagemajik.ai/artanalysis) to test your figure files. NAAS can convert your figure files to the TIFF file type and meet basic requirements (such as print size, resolution), or provide you with a report on issues that do not meet our requirements and that NAAS cannot fix. After uploading your figures to PLOS’s NAAS tool - https://ngplosjournals.pagemajik.ai/artanalysis, NAAS will process the files provided and display the results in the "Uploaded Files" section of the page as the processing is complete. If the uploaded figures meet our requirements (or NAAS is able to fix the files to meet our requirements), the figure will be marked as "fixed" above. If NAAS is unable to fix the files, a red "failed" label will appear above. When NAAS has confirmed that the figure files meet our requirements, please download the file via the download option, and include these NAAS processed figure files when submitting your revised manuscript. | N/A |
| --- | --- |
| **Reproducibility:** To enhance the reproducibility of your results, we recommend that authors of applicable studies deposit laboratory protocols in protocols.io, where a protocol can be assigned its own identifier (DOI) such that it can be cited independently in the future. Additionally, PLOS ONE offers an option to publish peer-reviewed clinical study protocols. Read more information on sharing protocols at https://plos.org/protocols?utm_medium=editorial-email&utm_source=authorletters&utm_campaign=protocols | N/A |
