# Supplementary material for: Exploring needs and priorities in digital health management for rare disease patients and their caregivers: A mixed-methods study
Source: PLOS Digit Health. 2026 Sep 28;5(9):e0001240. doi: 10.1371/journal.pdig.0001240 (PMC13618911; doi:10.1371/journal.pdig.0001240)
Supplement: Peer review history

# Peer review: Decision Letter 0

**DOI:** 10.1371/journal.pdig.0001240.r001

**Authors:** Leo Anthony Celi, John Robert Bautista

11 May 2026

Response to Reviewers Revised Manuscript with Track Changes Manuscript **Journal Requirements:**

1. Please amend your detailed Financial Disclosure statement. This is published with the article. It must therefore be completed in full sentences and contain the exact wording you wish to be published.

i. Please clarify all sources of financial support for your study. List the grants, grant numbers, and organizations that funded your study, including funding received from your institution. Please note that suppliers of material support, including research materials, should be recognized in the Acknowledgements section rather than in the Financial Disclosure.

ii. State the initials, alongside each funding source, of each author to receive each grant. For example: "This work was supported by the National Institutes of Health (####### to AM; ###### to CJ) and the National Science Foundation (###### to AM)."

iii. State what role the funders took in the study. If the funders had no role in your study, please state: “The funders had no role in study design, data collection and analysis, decision to publish, or preparation of the manuscript.”

iv. If any authors received a salary from any of your funders, please state which authors and which funders.

2. We note that your Data Availability Statement is currently as follows: The survey questionnaire and the discussion guide used for the focus groups are available as Supporting information files. All data generated or analyzed during this study are included in this published article.

Please confirm at this time whether or not your submission contains all raw data required to replicate the results of your study. Authors must share the “minimal data set” for their submission. PLOS defines the minimal data set to consist of the data required to replicate all study findings reported in the article, as well as related metadata and methods (https://journals.plos.org/plosone/s/data-availability#loc-minimal-data-set-definition).

For example, authors should submit the following data:

- The values behind the means, standard deviations and other measures reported;

- The values used to build graphs;

- The points extracted from images for analysis.

Authors do not need to submit their entire data set if only a portion of the data was used in the reported study.

If your submission does not contain these data, please either upload them as Supporting Information files or deposit them to a stable, public repository and provide us with the relevant URLs, DOIs, or accession numbers. For a list of recommended repositories, please see https://journals.plos.org/plosone/s/recommended-repositories.

If there are ethical or legal restrictions on sharing a de-identified data set, please explain them in detail (e.g., data contain potentially sensitive information, data are owned by a third-party organization, etc.) and who has imposed them (e.g., an ethics committee). Please also provide contact information for a data access committee, ethics committee, or other institutional body to which data requests may be sent. If data are owned by a third party, please indicate how others may request data access.

3. Please ensure that your Ethics Statement is available in its entirety at the beginning of your Methods section, under a subheading 'Ethics Statement'.

**Additional Editor Comments (if provided):** **Reviewers' Comments:**

**Comments to the Author**

1. Does this manuscript meet PLOS Digital Health’s publication criteria?

Reviewer #1: Yes

Reviewer #2: Partly

Reviewer #3: Partly

**********

2. Has the statistical analysis been performed appropriately and rigorously?

Reviewer #1: Yes

Reviewer #2: No

Reviewer #3: Yes

**********

3. Have the authors made all data underlying the findings in their manuscript fully available (please refer to the Data Availability Statement at the start of the manuscript PDF file)?

The PLOS Data policy

Reviewer #1: Yes

Reviewer #2: Yes

Reviewer #3: Yes

**********

4. Is the manuscript presented in an intelligible fashion and written in standard English?

Reviewer #1: Yes

Reviewer #2: Yes

Reviewer #3: Yes

**********

**Reviewer #1:**  Summary

The authors present a mixed-methods study (149 survey respondents, 15 focus group participants) analyzing what rare disease patients and caregivers want from digital health tools in Quebec. The community wants centralized data, symptom tracking, and better communication with clinicians. The paper shows why generic health apps do not work for this population.

In my comments below, I make some suggestions of what to include. The authors are free to include them or not, the intention is to be helpful not to constrain them to a particular view. I hope that they are useful because I think this should be accepted/published, subject to minor changes.

Major

1. Sample Bias and Response Rates: The 90% female sample is expected, but warrants brief acknowledgement and discussion, because it's socially important. Sociologically, women carry a disproportionate caregiving burden for complex needs, and female patients generally experience longer diagnostic odysseys, often facing "medical gaslighting". This drives them to advocacy groups like RQMO. The authors should state how these systemic drivers create bias, and note how it affects findings on digital literacy or trust. Do the authors have a response rate? Knowing whether 149 respondents came from 300 or 3,000 invitations would clarify the engagement level.

2. Alert Fatigue and Behavioral Economics: The mention of "alert fatigue" (Lines 688-691) identifies a common failure point. From a behavioral economics perspective, clinicians facing a flood of raw, patient-generated symptom data will discount or ignore it due to cognitive overload. This friction prevents digital health adoption. Patient-centered tools only work if they are aggressively filtered to be clinician-friendly. The discussion needs to reflect this.

3. Privacy and Self-Selection: The conclusion that rare disease patients are willing to share data (only 50% expressed privacy concerns) is likely overstated. Recruiting through advocacy networks yields a highly activated cohort desperate for solutions. This group self-selects for higher trust in data sharing compared to the general population. I'm not arguing with the authors, and this is my view. If the authors think differently, it might be worth suggesting something like, "a reviewer suggested X, however...". What that does is demonstrate that there isn't consensus in the interpretation and in my view, adds value as a potential avenue for further exploration. For what it's worth, I reckon an discrete choice experiment could be really useful and interesting to undertake here, and although I'm not a native French-speaker, if the authors are interested in further advice/suggestions on this, I'm happy to provide them.

4. Generalizability (The Quebec Context): Quebec's RAMQ system provides universal public insurance but struggles with geographic bottlenecks and wait times (common to many social health systems). Patients are fighting systemic access barriers rather than financial ones. The findings generalize best to similar universal, geographically dispersed systems--such as the UK NHS, Australia's Medicare, or New Zealand-- rather than multi-payer environments like the US. This important, because under-recognised is both access difficulties and catastrophic health expenditure for people with rare diseases.

Minor

- Line 284: The qualitative quote mentioning "20-year wait times" is compelling. Mention this in the introduction to frame why the diagnostic odyssey requires digital intervention. I'm tempted to provide a quote to one of my own papers here, but on principle I won't There's plenty of great papers describing wait times. The typically quoted figure is somewhere around years. I don't know how to interpret the "20-year" experience, but I feel like it might be important in the future for us to come back to this.

- Line 364 (AI): Participants suggesting AI for symptom pattern recognition is topical. Briefly discuss how patients currently use LLMs for self-diagnosis, and the clinical risks involved. You might (I'm not sure), identify a gap in the literature around this. This group might be well placed to research that, but in either case, it signposts for future researchers, making this paper an important citation in work like this.

Conclusion

This is a strong paper providing useful data in the digital health space. Hopefully it's evidence that I enjoyed it and I appreciate that the work was done. The mixed-methods approach captures the specific frustrations of the rare disease community. Addressing the drivers of demographic bias, adjusting the privacy findings, and expanding on the behavioral friction of clinician alert fatigue will improve the manuscript. I recommend minor revision.

Note to the editorial team: If accepted, I advise promoting this piece. It will draw attention from pharma/lifesciences groups, the rare disease community, and organizations like the World Economic Forum, which have an active interest in rare disease support and digital management.

Reporting Checklist Assessment (COREQ)

Recommendation: The authors should review COREQ or SRQR standards and clarify the missing qualitative methodology details (particularly saturation, non-participation, and participant checking). If you download either/both from Equator, assess against this, implement the improvements, then include the checklist as an attachment. They can be incorporated into a supplement and don't need to be in the manuscript. What this does is increase the assessed quality, particularly for things like meta-analyses, and this increases the likelihood of citation/impact. I suspect (my view), that increasingly as AI-assisted manuscript review comes in, that checklists will be considered standard. I've asked for them for many years, but this context makes it even more important and worth the additional work.

An cursory independent assessment against the 32-item COREQ checklist shows:

- Reported: Methodological orientation, participant selection via advocacy groups, setting (online), sample demographics, data coder count, and quotation presentation.

- Missing/Incomplete:

-- Research Team: Specific credentials, occupation, and gender of the facilitator; participant knowledge of the interviewer.

-- Study Design: Data saturation, rates of non-participation/drop-outs for the focus groups, and whether transcripts were returned to participants.

-- Analysis: Formal participant checking of the findings or quotes.

**Reviewer #2:**  This study examines the role of digital health technologies in addressing the complex challenges faced by individuals living with rare diseases and their caregivers, including barriers to diagnosis, treatment access, and care coordination. The study addresses a highly relevant and underexplored area, and its mixed-methods approach has the potential to provide valuable, practice-oriented insights into the needs and priorities of rare disease patients and their caregivers.

That said, there are several areas where further clarification and elaboration would strengthen the manuscript and enhance its overall contribution:

In the methods section, the authors state that RQMO members were recruited as participants. However, readers who are not familiar with this organization may lack sufficient background to understand the sampling choice. It would be helpful to briefly describe what RQMO is, the characteristics of its members, and the rationale for targeting this group. In addition, the authors should discuss the strengths and limitations associated with this sampling strategy.

Based on the data analysis and results sections, the quantitative survey appears to be used primarily for descriptive purposes, which somewhat limits the implications of the findings. Given the context of rare disease patients and their caregivers, one might expect more exploratory insights into their motivations and needs regarding telemedicine or digital health tools. However, participants were restricted to selecting from predefined options, which may have constrained the depth of responses. This raises a broader question about how the sequential mixed-methods design was intended to function. Specifically, what aspects were meant to be addressed by the survey, and what additional insights were expected from the interviews? The complementary roles of the two methods should be clarified.

It is also unclear why caregivers were asked to respond from the perspective of the individuals they care for, rather than from their own viewpoints. Caregivers may have distinct needs and experiences, and capturing these directly could provide valuable insights. The authors should justify this design choice.

Finally, the importance of telemedicine for rare disease patients and their caregivers could be better articulated. How does telemedicine use in this population differ from what has been reported in the broader literature on general patient populations? A more explicit comparison in both the introduction and discussion sections would help to strengthen the study’s contribution.

I wish the authors the best in further refining and developing this work for publication.

**Reviewer #3:**  Understanding the challenge of consolidating data from rare diseases, I highy commend the efforts undertaken by the team. While the dataset is small (n = 15 for the followup activities), the 150+ respondents can compensate for this.

**********

what does this mean?). If published, this will include your full peer review and any attached files.

**Do you want your identity to be public for this peer review?**  If you choose “no”, your identity will remain anonymous but your review may still be made public.

For information about this choice, including consent withdrawal, please see our Privacy Policy

Reviewer #1: No

Reviewer #2: No

Reviewer #3: No

**********

**Figure resubmission:**

**Reproducibility:**

To enhance the reproducibility of your results, we recommend that authors of applicable studies deposit laboratory protocols in protocols.io, where a protocol can be assigned its own identifier (DOI) such that it can be cited independently in the future. Additionally, PLOS ONE offers an option to publish peer-reviewed clinical study protocols. Read more information on sharing protocols at https://plos.org/protocols?utm_medium=editorial-email&utm_source=authorletters&utm_campaign=protocols

# Author comment: Author response to Decision Letter 1

**DOI:** 10.1371/journal.pdig.0001240.r002

4 Jul 2026

Attachment Submitted filename: Response to reviewers_v2026-07-04.docx

# Peer review: Decision Letter 1

**DOI:** 10.1371/journal.pdig.0001240.r003

**Authors:** Leo Anthony Celi

8 Sep 2026

Exploring Needs and Priorities in Digital Health Management for Rare Disease Patients and their Caregivers: A Mixed-Methods Study

PDIG-D-26-00150R1

Dear Dr Ethier,

We are pleased to inform you that your manuscript 'Exploring Needs and Priorities in Digital Health Management for Rare Disease Patients and their Caregivers: A Mixed-Methods Study' has been provisionally accepted for publication in PLOS Digital Health.

Before your manuscript can be formally accepted you will need to complete some formatting changes, which you will receive in a follow-up email from a member of our team.

Please note that your manuscript will not be scheduled for publication until you have made the required changes, so a swift response is appreciated.

IMPORTANT: The editorial review process is now complete. PLOS will only permit corrections to spelling, formatting or significant scientific errors from this point onwards. Requests for major changes, or any which affect the scientific understanding of your work, will cause delays to the publication date of your manuscript.

If your institution or institutions have a press office, please notify them about your upcoming paper to help maximize its impact. If they'll be preparing press materials, please inform our press team as soon as possible -- no later than 48 hours after receiving the formal acceptance. Your manuscript will remain under strict press embargo until 2 pm Eastern Time on the date of publication. For more information, please contact digitalhealth@plos.org.

Thank you again for supporting Open Access publishing; we are looking forward to publishing your work in PLOS Digital Health.

Best regards,

Leo Anthony Celi, MD MS MPH

Editor-In-Chief

PLOS Digital Health

***********************************************************

**Additional Editor Comments (if provided):**

**Reviewer Comments (if any, and for reference):**

Reviewer's Responses to Questions

**Comments to the Author**

Reviewer #2: All comments have been addressed

**********

publication criteria?

Reviewer #2: Yes

**********

3. Has the statistical analysis been performed appropriately and rigorously?

Reviewer #2: Yes

**********

4. Have the authors made all data underlying the findings in their manuscript fully available (please refer to the Data Availability Statement at the start of the manuscript PDF file)?

The PLOS Data policy

Reviewer #2: No

**********

5. Is the manuscript presented in an intelligible fashion and written in standard English?

Reviewer #2: Yes

**********

Reviewer #2: The authors have thoroughly addressed my comments, and I sincerely appreciate their efforts in revising the manuscript; I wish them all the best with this project and their future work.

**********

what does this mean?). If published, this will include your full peer review and any attached files.

**Do you want your identity to be public for this peer review?**  If you choose “no”, your identity will remain anonymous but your review may still be made public.

For information about this choice, including consent withdrawal, please see our Privacy Policy

Reviewer #2: No

**********
